# Hydrogen sulfide attenuates spatial memory impairment and hippocampal neuroinflammation in beta-amyloid rat model of Alzheimer’s disease

**DOI:** 10.1186/1742-2094-9-202

**Published:** 2012-08-17

**Authors:** Aiguo Xuan, Dahong Long, Jianhua Li, Weidong Ji, Meng Zhang, Lepeng Hong, Jihong Liu

**Affiliations:** 1 Department of Anatomy, Guangzhou Medical University, Guangzhou, China; 2 Department of Physiology, Guangzhou Medical University, Guangzhou, China; 3 Department of Urology, Minimally Invasive Surgery Center, Guangdong Provincial Key Laboratory of Urology, The First Affiliated Hospital of Guangzhou Medical University, Guangzhou, China; 4 Department of Neurobiology, Southern Medical University, Guangzhou, China

**Keywords:** Alzheimer’s disease, Amyloid-β, Neuroinflammation, Hydrogen sulfide, p38 mitogen-activated protein kinase, p65 nuclear factor-κB

## Abstract

**Background:**

Endogenously produced hydrogen sulfide (H_2_S) may have multiple functions in brain. An increasing number of studies have demonstrated its anti-inflammatory effects. In the present study, we investigated the effect of sodium hydrosulfide (NaHS, a H_2_S donor) on cognitive impairment and neuroinflammatory changes induced by injections of Amyloid-β_1-40_ (Aβ_1-40_), and explored possible mechanisms of action.

**Methods:**

We injected Aβ_1-40_ into the hippocampus of rats to mimic rat model of Alzheimer’s disease (AD). Morris water maze was used to detect the cognitive function. Terminal deoxynucleotidyl transferase-mediated dUTP nick end labeling (TUNEL) assay was performed to detect neuronal apoptosis. Immunohistochemistry analyzed the response of glia. The expression of interleukin (IL)-1β and tumor necrosis factor (TNF)-α was measured by enzyme-linked immunosorbent assay (ELISA) and quantitative real-time polymerase chain reaction (qRT-PCR). The expression of Aβ_1-40_, phospho-p38 mitogen-activated protein kinase (MAPK), phospho-p65 Nuclear factor (NF)-κB, and phospho-c-Jun N-terminal Kinase (JNK) was analyzed by western blot.

**Results:**

We demonstrated that pretreatment with NaHS ameliorated learning and memory deficits in an Aβ_1-40_ rat model of AD. NaHS treatment suppressed Aβ_1-40_-induced apoptosis in the CA1 subfield of the hippocampus. Moreover, the over-expression in IL-1β and TNF-α as well as the extensive astrogliosis and microgliosis in the hippocampus induced by Aβ_1-40_ were significantly reduced following administration of NaHS. Concomitantly, treatment with NaHS alleviated the levels of p38 MAPK and p65 NF-κB phosphorylation but not JNK phosphorylation that occurred in the Aβ_1-40_-injected hippocampus.

**Conclusions:**

These results indicate that NaHS could significantly ameliorate Aβ_1-40_-induced spatial learning and memory impairment, apoptosis, and neuroinflammation at least in part via the inhibition of p38 MAPK and p65 NF-κB activity, suggesting that administration of NaHS could provide a therapeutic approach for AD.

## Background

Alzheimer’s disease (AD) is a devastating and progressive neurodegenerative disorder characterized by extracellular deposition of Amyloid-β (Aβ) protein and intraneuronal neurofibrillary tangles (NFTs). Microglia have been implicated in the progressive nature of numerous neurodegenerative or neuroinflammatory diseases such as AD [1]. The premise of deleterious microglial activation in AD has been supported by analysis of postmortem brains of patients with AD [2], where microglial over-activation occurred before neuropil damage, suggesting that it plays a causal role in the development of AD. The core of the senile plaque is the deposition of Aβ and the activated microglia and astroglia are around the senile plaque. In these glias, numerous inflammation factors including interleukin-1β (IL-1β), interleukin-6 (IL-6), tumor necrosis factor-α (TNF-α) and inducible nitric oxide synthase (iNOS), and cyclooxygenase-2 (COX-2), and so on, are overexpressed [3,4]. Accumulating evidence indicates that neuroinflammatory processes may contribute to the pathophysiology of AD. However, traditional anti-inflammatory therapies such as non-steroidal anti-inflammatory drugs (NSAIDs) have produced mixed and conflicting results [5], highlighting the need for new and more specific anti-inflammatory targets.

Hydrogen sulfide (H_2_S) is best known as a poisonous gas with an extremely unpleasant odor. It is endogenously produced in the brain from cysteine by cystathionine β-synthase (CBS) and cystathione γ-lyase (CGL) [6]. CBS is the main H_2_S producing enzyme in brain. A recent study showed that 3-mercaptopyruvate sulfurtransferase (3MST) in combination with cysteine aminotransferase (CAT) also produces H_2_S from cysteine. In addition, 3MST is localized to neurons, and the levels of bound sulfane sulfur, the precursor of H_2_S, are greatly increased in the cells expressing 3MST and CAT but not increased in cells expressing functionally defective mutant enzymes [7]. Numerous studies showed that H_2_S has anti-oxidant, anti-apoptotic effects in neuron and glial cells [8,9]. H_2_S induces alterations in Ca^2+^ in astrocytes and microglia [10,11], suggesting it may have anti-inflammatory properties. H_2_S produces an anti-inflammatory effect in lipopolysaccharide (LPS)-induced inflammation in both primary cultured microglia and immortalized murine BV-2 microglial cells [12]. The levels of S-adenosylmethionine (SAM), an activator of CBS, are lower in AD brains than that in the brains of normal individuals [13]. Another study showed that H_2_S is an endogenous anti-inflammatory and neuroprotective agent, and H_2_S releasing drugs may have therapeutic potential in neurodegenerative disorders of aging such as AD and Parkinson’s disease (PD) [14].

The above-mentioned roles of H_2_S raise the possibility that H_2_S may be associated with the pathology of AD. However, so far, a possible role for H_2_S as an anti-inflammatory agent in rat model of AD has not been extensively evaluated. The focus of the present study was to elucidate the effect of NaHS on cognitive impairment and neuroinflammatory changes in rat model of AD as well as possible mechanisms of action.

## Methods

### Surgery and drug administration

Healthy male Wistar rats (220 to 250 g) were randomly divided into four groups (*n* = 56 for each group): sham (control) group, sham + NaHS group, Aβ_1-40_ group, and Aβ_1-40_ + NaHS group. Rats in the Aβ_1-40_ + NaHS group were administered with NaHS (Sigma, USA) by intraperitoneal injection (i.p.) at a dose of 5 mg/kg once daily, 3 days before surgery and thereafter continuously for 9 days[15,16]. Three days after treatment with NaHS, anesthesia was induced by chloral hydrate (35 mg/100 g weight, i.p.). Aβ_1-40_ (10 nmol in 10 μL of sterile PBS) was incubated at 37°C for 1 week to induce the aggregation of Aβ_1-40_[17]. The aggregated Aβ_1-40_ and vehicle was injected slowly over 10 min into the right dentate gyrus (DG) of rats at the following coordinates: anterior/posterior −3.3 mm, media/lateral 2.0 mm, and dorsal/ventral −3.3 mm ventral to the skull surface. The rectal temperature was maintained at 36°C to 37°C for all animals throughout the experiment. All animal experiments were performed according to protocols approved by the local animal care committee.

### Morris water maze

The Morris water maze task was evaluated as previously described [18] with slight modification. Trials were performed during days 8 to 11 after the injection of Aβ_1-40_. The task for all of the animals in each trial consisted of finding a hidden clear plastic platform (10 cm diameter) that was placed 50 cm away from the wall of the water maze (150 cm in diameter, 60 cm in depth) and 1 cm below the water. The platform remained in the same position for all trials and sessions. The starting quadrant was randomized every day, with all animals using the same order. The animals were faced towards the pool wall before being released. The time required to reach the hidden platform and the swimming speed were recorded. The animals were allowed to rest 30 s on the platform between trials. If an animal failed to reach the platform in 120 s, it was manually guided to the platform. Each animal underwent two sessions (each contains four trials: NE; NW; SE; SW) per day for 4 consecutive days. Before surgery, animals were screened (without platform in the pool) for any rats that could not swim. Seven days after the injection of Aβ_1-40_, animals were examined as above.

### Immunohistochemistry and TUNEL staining

Immunohistochemistry was performed on the eighth day after Aβ_1-40_ injection. After being microwaved for 5 min and washed three times in PBS (pH 7.4), sections were successively incubated with 0.3% H_2_O_2_ in methanol for 10 min, 10% normal goat serum in PBS for 20 min, and primary antibody (anti-GFAP, anti-OX42, Millipore, USA) dissolved in 2% normal goat serum, 0.3% Triton X-100, 0.05% NaN_3_ in PBS at 4°C overnight, and then 37°C for 30 min. After rinsing three times in PBS, the sections were incubated with biotinylated anti-mouse or anti-rabbit secondary antibodies (Boster, China) in PBS for 30 min at 37°C, then incubated with avidin-biotin-peroxidase solution (SABC kit, Boster, China) and colorized with a DAB kit (Boster, China).

To detect cells undergoing apoptosis, TUNEL technique was performed according to the manufacturer’s protocol supplied within the *in situ* Cell Death Detection Kit. The sections were immersed in 3% H_2_O_2_ for inactivation of endogenous hydrogen peroxidase activity. After rinsing with PBS, the sections were incubated with proteinase K solution at 37°C for 20 min to enhance the permeability. Then they were incubated for 60 min at 37°C with TUNEL reaction mixture and again incubated for 30 min at 37°C with converter-POD. The sections were rinsed in PBS, incubated for 10 min with DAB substrate solution and rinsed again with PBS. Counter staining was done with 0.5% methyl green. Positive and negative controls were carried out on slides from the same block. For TUNEL staining, 10 fields were chosen from each group and the percent of TUNEL-positive cells were calculated according to this relation: % TUNEL-positive neurons = (TUNEL-positive neurons (brown)/TUNEL-positive neurons (brown) + normal neurons (green)) × 100.

### Measurement of pro-inflammatory cytokines

Hippocampal samples were homogenized in 10 wet weight volumes of TBS, pH 8.0, containing a cocktail of protease inhibitors (20 mg/mL each of pepstatin A, aprotinin, phosphoramidon, and leupeptin, 0.5 mM PMSF, and 1 mM EGTA). Samples were sonicated briefly (10 W, 2 × 5 s) and centrifuged at 100,000 × g for 20 min at 4°C. The soluble fraction (supernatant) was used for IL-1β and TNF-α ELISAs (R&D Systems, USA).

### Real time RT-PCR analysis

Expressions of genes were further confirmed by real time PCR. Total RNA from hippocampus tissues were extracted using TriZol reagent (Invitrogen). Reverse transcription was performed with an ExScript RT Reagent Kit (Takara Bio Inc., China). Real-time PCR analysis was undertaken using SYBR Premix Ex Taq (Takara Bio Inc., China). The primers sequences for IL-1β were 5′-GCT GTG GCA GCT ACC TAT GTC TTG-3′ (sense) and 5′-AGG TCG TCA TCA TCC CAC GAG-3′ (antisense). The primer sequences for TNF-α were 5′-GTG ATC GGT CCC AAC AAG GA-3′ (sense) and 5′-CTC CCA CCC TAC TTT GCT TGT G-3′ (antisense). The primer sequences for β-actin were 5′-TGA CAG G TG CAG AAG GAG A-3′ (sense) and 5′-TAG AGC CAC CAA TCC ACA CA-3′ (antisense). The real-time PCR conditions were as follows: initial denaturation at 95°C for 10 s followed by 39 cycles of 95°C for 5 s and 60°C for 20 s. The expression levels of the genes were quantified by comparison with a standard curve and normalized relative to levels of ß-actin.

### Western blot analysis

Expression of Aβ_1-40_, phospho-p38 MAPK, phospho-p65 NF-κB, and phospho-JNK was analyzed by western blot. Thirty μg protein of each sample was heated at 100°C for 5 min with a loading buffer containing 0.125 M Tris–HCl (pH 6.8), 20% glycerol, 4% SDS, 10% mercaptoethanol, and 0.002% bromophenol blue. It was then separated by sodium dodecyl sulfate-polyacrylamide gel electrophoresis (SDS-PAGE) using 10% acrylamide gels. The proteins were transferred onto PVDF membranes (pore size, 0.45 μm). Blotting membranes were incubated with 3% bovine serum albumin (BSA) in tris buffered saline with tween (TBST) (10 mmol/L Tris (pH 7.5), 150 mmol/L NaCl, 0.05% Tween-20) and probed with corresponding primary antibodies (anti-Aβ_1-40_, anti-phospho-p65 NF-κB, anti-phospho-p38 MAPK, and anti-phospho-JNK, CST, USA) at 4°C overnight. After incubation with horseradish peroxidase-coupled secondary antibodies for 2 h at room temperature, bands were quantitated by densitometry (UVP Upland, CA).

### Statistical analysis

All values were expressed as the mean ± standard error of the mean (SEM). For the behavioral experiments, the escape latency during the training tests was determined by two-way repeated factor analysis of variance (ANOVA) with Student-Newman-Keuls tests. All other assessments were analyzed using a one-way ANOVA followed by Student-Newman-Keul’s or Dunnett’s *post-hoc* analysis. In all cases, *P* < 0.05 was considered significant.

## Results

### NaHS prevented Aβ-induced impairment of spatial learning

**Figure 1:**
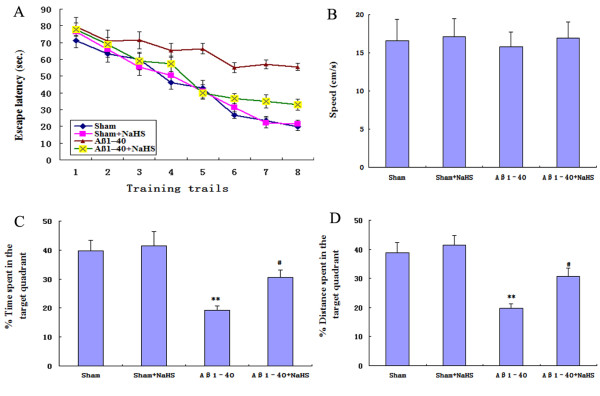
**Effect of NaHS on Aβ**_**1-40**_**-induced cognitive impairment.** Aβ_1-40_ was slowly injected into the hippocampus, and subjected to the Morris water maze test 7 days later. (**A**) The escape latency in the navigation test. (**B**) The average swim speed among the groups. (**C**) The percent of (%) time in the targeted quadrant where the platform had been located in the spatial exploring test. (**D**) The percent of (%) distance in the targeted quadrant where the platform had been located in the spatial exploring test. Data presented as mean ± S.E.M., *n* = 10-13. **P < 0.01 *vs.* sham; ^#^*P* < 0.05 *vs.* Aβ_1-40._

To investigate whether the pre-treatment with NaHS led to functional improvement, we employed the Morris water maze task to examine hippocampus-involved learning and memory. All animals were able to swim normally and find the hidden platform during the training trials. After being trained twice per day for two consecutive sessions, sham and sham + NaHS rats were able to reach the hidden platform in a shorter time during the training (Figure 1A). However, the learning and memory abilities of Aβ_1-40_-injected rats were significantly impaired compared with the sham group (*P* < 0.01) (Figure 1A). A significant decrease in escape latency was observed in the NaHS + Aβ_1-40_ group compared with the Aβ_1-40_-injected group (*P* < 0.01) (Figure 1A). There was no significant difference in average swim speed among the groups (Figure 1B). These results clearly indicate that NaHS treatment significantly ameliorated severe deficiencies in spatial cognitive performance induced by Aβ_1-40_.

In the probe trial of the Morris water maze test, Aβ_1-40_ had a significant effect on the time and distance in target quadrant compared with the sham group (*P* < 0.01). Compared with the Aβ_1-40_-injected group, NaHS + Aβ_1-40_ rats displayed more time and distance swimming in the target quadrant (*P* < 0.05) (Figure 1C, D).

### NaHS suppressed Aβ_1-40_-induced apoptosis in Aβ_1-40_-injection rat model

**Figure 2.**
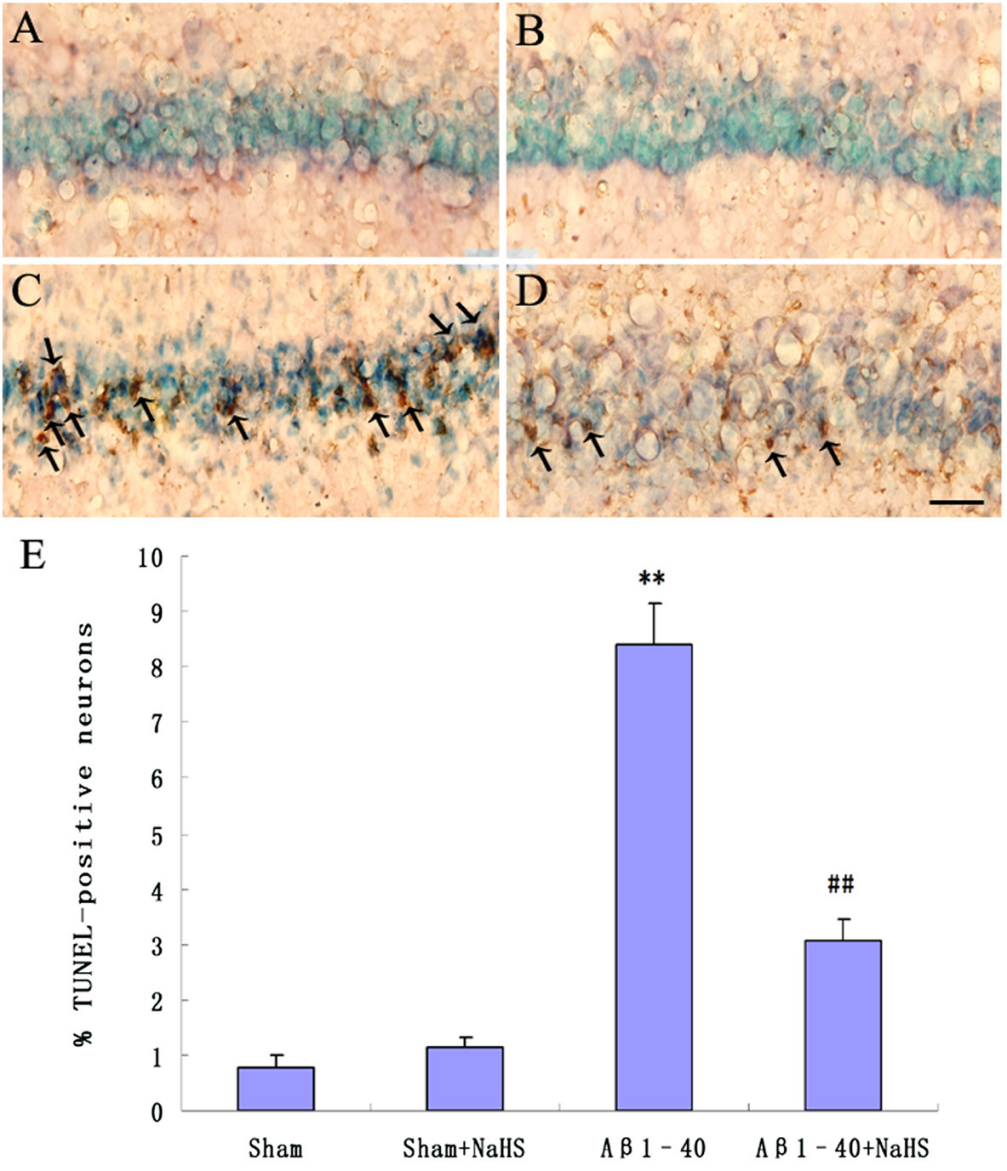
**Effects of NaHS on DNA fragmentation in the hippocampus of rats injected with Aβ**_**1-40**_**.** (**A, B**) No TUNEL-reactive cell was detected in the hippocampus from sham and sham + NaHS rats. (**C**) The significant number of degenerating pyramidal neurons, labeled with the TUNEL technique was observed in the CA1 subfield of the hippocampus. (**D**) The number of TUNEL-positive neurons significantly decreased in the hippocampus from Aβ_1-40_-injected rats receiving NaHS. Scale bar: 40 μm. Arrows point to degenerating pyramidal neurons with their nuclei stained with TUNEL technique. (**E**) Quantitative analysis of % TUNEL-positive neurons. Data are given as mean ± S.E.M.. **P < 0.01 *vs.* sham; ^##^*P* < 0.01 *vs.* Aβ_1-40._

To confirm the protective effect of NaHS on Aβ_1-40_-induced apoptosis, sections through the hippocampus were also examined for the presence of fragmented DNA via TUNEL assay. Microscopic inspection of the hippocampal sections from sham and sham + NaHS rats revealed morphologically normal neurons with no TUNEL reaction. After the injection of Aβ_1-40_, a significant number of TUNEL-positive pyramidal neurons with different degrees of DNA fragmentation were detected in the CA1 subfield of the hippocampus (*P* < 0.01 *vs.* sham). Treatment with NaHS significantly reduced the number of TUNEL-positive neurons (*P* < 0.01 *vs.* Aβ_1-40_) (Figure 2A-E).

### NaHS lowered protein levels of Aβ_1-40_ in the hippocampus of rats

**Figure 3:**
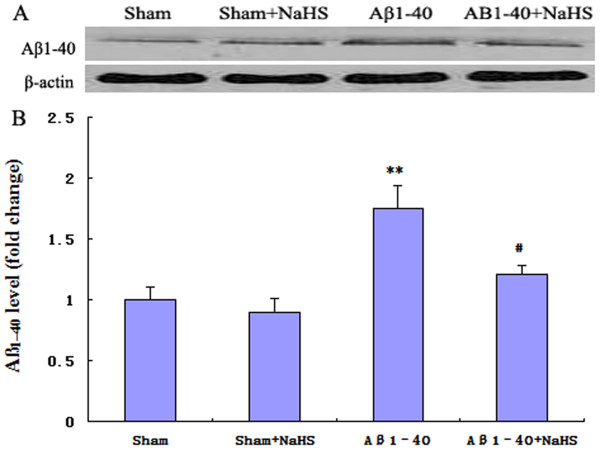
**NaHS pre-treatment reduces Aβ**_**1-40**_**levels.** Aβ_1-40_ rats were treated with NaHS (5 mg/kg i.p. once daily). (**A**) Western blot of Aβ_1-40_ protein contents. In hippocampal area CA1 the basal levels of Aβ_1-40_ peptides decreased significantly after NaHS treatment. (**B**) The relative optical density was normalized to β-Actin. Results are expressed as mean ± S.E.M. with five rats in each group. **P < 0.01 *vs.* sham; ^#^*P* < 0.05 *vs.* Aβ_1-40._

Progressive accumulation of Aβ peptides are a major factor in the development of AD pathogenesis [19]. Immunoblot analysis was used to assess the effect of NaHS on levels of Aβ_1-40_ in area CA1. Infusion of Aβ peptides in normal rats resulted in a marked (*P* < 0.01) accumulation of Aβ_1-40_ levels. NaHS treatment significantly decreased levels of Aβ_1-40_, in NaHS + Aβ_1-40_ rats by approximately 31%, compared to those in Aβ rats (Aβ: 1.75 ± 0.19; NaHS + Aβ_1-40_:1.21 ± 0.07, *n* = 5 rats/group) (Figure 3A, B).

### NaHS decreased Aβ-induced astrocytic and microglial response

**Figure 4:**
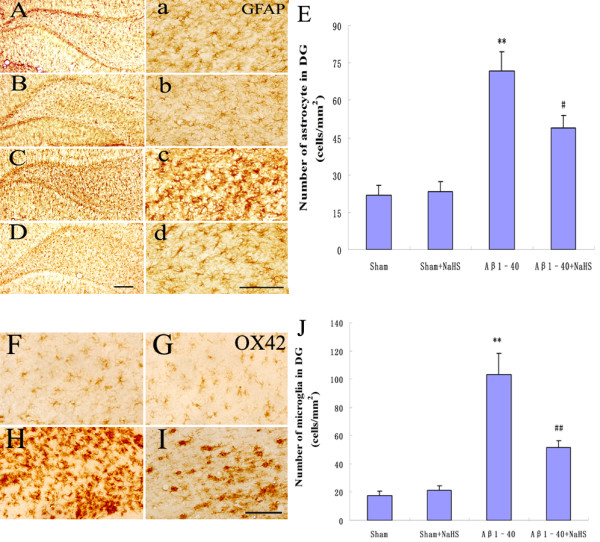
**Effect of NaHS on Aβ**_**1–40**_**-induced the activation of glia in the DG region of rat hippocampus.** (**A-E, a-d**) The distribution and number of GFAP-immunoreactive astrocytes in sham, sham + NaHS, Aβ_1-40_, and NaHS + Aβ_1-40_ rats. Scale bar, 500 μm (**A-D**) and 50 μm (**a-d**). (**F-J**) Representative photographs and number of OX-42-immunopositive cells in sham, sham + NaHS, Aβ_1-40_, and NaHS + Aβ_1-40_ rats. Scale bar: 250 μm. Six tissue sections per rat were used for the analysis (*n* = 8-10). Data are presented as the mean ± S.E.M. **P < 0.01 *vs.* sham; ^##^*P* < 0.01, ^#^*P* < 0.05 *vs.* Aβ_1-40._

Intrahippocampal injection of Aβ oligomers has been shown to have extended neuroinflammatory responses displaying a significant increase in astrocytic and microglial response that is associated with age and amyloid deposition [20,21]. To evaluate the effect of NaHS on the glial response, we performed GFAP (astrocytic) and OX42 (microglial) staining in the hippocampus. GFAP immunostaining demonstrated that injection of Aβ_1-40_ caused reactive gliosis as demonstrated by upregulation of GFAP expression and the presence of hypertrophic astrocytes in the hippocampus (*P* < 0.01) (Figures 4A-E and 4a-d). In the NaHS + Aβ_1-40_ group the number of GFAP-immunoreactive astrocytes was significantly reduced compared to the Aβ_1-40_-injected group (*P* < 0.05) (Figures 4A-E and 4a-d). A similar effect was exerted in the NaHS + Aβ_1-40_ group where the intensity of OX42-positive microglia was significantly reduced compared to the Aβ_1-40_-injected group (Figure 4F-J).

### NaHS attenuated Aβ-induced increases in the levels of cytokine production and mRNA in the hippocampus

**Figure 5:**
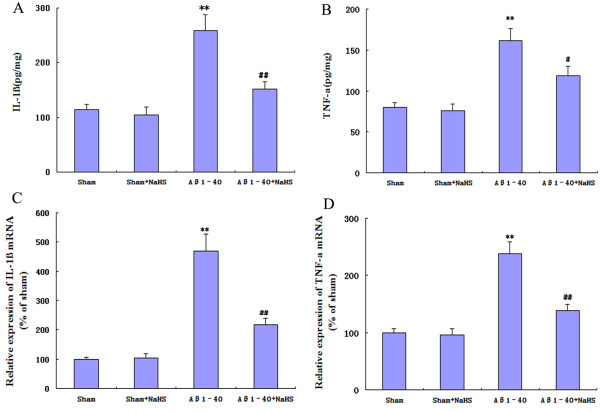
**Effect of NaHS on the IL-1β, TNF-α production, and mRNA expressions.** IL-1β and TNF-α levels in the hippocampus were measured via ELISA. The expressions of IL-1β and TNF-α mRNA in the hippocampus were detected by real time RT-PCR. Aβ_1-40_ injection into the hippocampus significantly increased the IL-1β, TNF-α production, and mRNA expressions. Treatments with NaHS significantly decreased the levels and mRNA over-expressions of IL-1β and TNF-α. (**A**) The level of IL-1β. (**B**) The level of TNF-α. (**C**) The expressions of IL-1β mRNA. (**D**) The expressions of TNF-α mRNA. Data are mean ± S.E.M., *n* = 4. **P < 0.01 *vs.* sham; ^##^*P* < 0.01, ^#^*P* < 0.05 *vs.* Aβ_1-40._

We next addressed whether NaHS was able to ameliorate a generalized pro-inflammatory response from glia. The pro-inflammatory cytokines response was tested by measuring levels of IL-1β and TNF-α in hippocampal brain homogenates. Aβ_1-40_ injection significantly increased the levels of IL-1β and TNF-α, and NaHS was able to significantly reduce this response, although levels did not return to that of control (Figure 5A, B).

The effects of NaHS on the mRNA of IL-1β and TNF-α were investigated by real-time RT-PCR. Compared with the sham group, injection of Aβ_1-40_ in the hippocampus highly increased the mRNA of IL-1β and TNF-α, which were approximately 4.7-fold and 2.4-fold, respectively (*P* < 0.01, Figure 5C, D). However, compared to the Aβ_1-40_ group, treatment with NaHS significantly decreased the mRNA expressions of these selected genes (*P* < 0.01, Figure 5C, D).

### NaHS decreased the activation of phospho-p38 MAPK and phospho-p65 NFκB, but not phospho-JNK in the hippocampus

**Figure 6:**
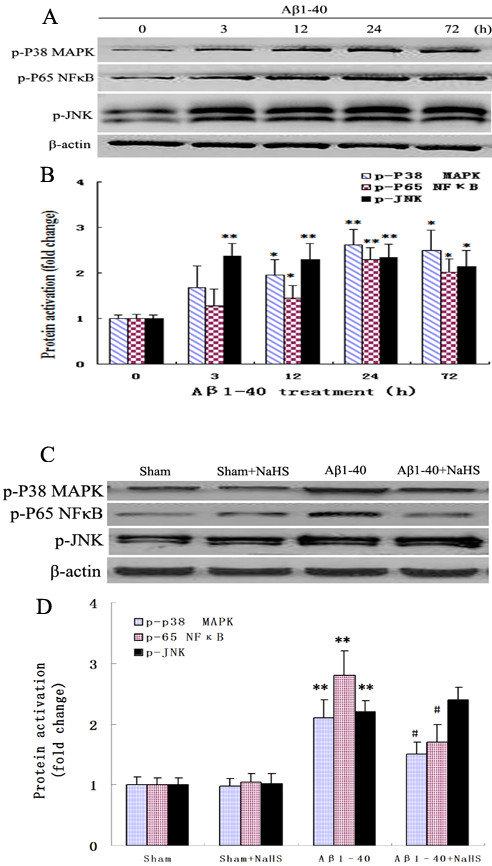
**Western blotting analysis of the relative protein contents.** (**A, C**) Western blot of various protein contents for phospho-p38 MAPK, phospho-p65 NFκB, and phospho-JNK. (**B, D**) The relative optical density was normalized to β-Actin. Data are mean ± S.E.M., *n* = 4. **P < 0.01 *P < 0.05 *vs.* sham(control); ^#^*P* < 0.05 *vs.* Aβ_1-40._

To further explore the molecular mechanisms underlying the inhibitory effect of NaHS on the expressions of IL-1β and TNF-α, the expressions of phospho-p38 MAPK, phospho-p65 NFκB, and phospho-JNK were determined by western blot analysis. Aβ_1-40_ injection into the hippocampus significantly enhanced the p38 MAPK, p65 NF-κB, and JNK phosphorylation (*P* < 0.01, Figure 6A, B). However, treatment with NaHS caused a significant decrease in the phosphorylation of p38 MAPK and p65 NF-κB but not JNK (*P* < 0.05, Figures 6C, D). Our results show that NaHS treatment suppressed Aβ_1-40_-induced activation of p38 MAPK and p65 NF-κB but not JNK, which may contribute to the inhibition of NaHS on the Aβ_1-40_-induced IL-1β and TNF-α production.

## Discussion

The deposition of Aβ in brain areas involved in cognitive functions is assumed to initiate a pathological cascade that results in synaptic dysfunction, synaptic loss, and neuronal death [22]. It has been proposed that Aβ_1-40_ aggregates play an important role in the pathogenesis of AD [23]. Numerous reports have showed that the injection of Aβ_1-40_ into rat hippocampus provides an effective model to mimic some of the pathologic and behavioral changes of AD [24-26]. In our study, we demonstrated that Aβ_1-40_ injection could induce memory deficits and NaHS treatment could also effectively ameliorate Aβ-induced impairment of spatial learning.

Memory impairment in Aβ-injected rats was associated with a significant reduction in apoptosis. Apoptosis has been consistently implicated in Aβ-induced neuronal damage *in vitro*, in animal models of AD, and also postmortem studies of AD brain [27-29]. The mechanisms underlying Aβ-mediated neurotoxicity still remain to be elucidated, but mounting evidence suggests the involvement of Aβ-induced neuroinflammatory in the disease process with AD. Studies have shown that Aβ induces the production of neuroinflammatory molecules, which may contribute to the pathogenesis of numerous neurodegenerative diseases [30-32]. However, lots of studies have also demonstrated that anti-inflammatory compounds could exhibit neuroprotective effects in damaged brain cells [33-35]. Central administration of NaHS prevented Aβ_1-40_-evoked apoptosis. Decrease in TUNEL-positive neurons may stem from its general suppression of the neuroinflammatory context in the Aβ_1-40_-inflicted hippocampus, thus attenuating the inflammatory cell death.

Evidence suggests that inflammatory reaction induced by Aβ in the AD involves astrogliosis, microgliosis, cytokine elevation, and changes in acute phase proteins [22,36]. Activated astrocytes and microglia can secrete inflammatory cytokines and mediators that promote the formation of Aβ and NFTs through numerous signal transduction pathways [37]. In the brains of AD rats, the production of IL-1β and TNF-α play an important role in augmenting inflammatory reaction and formation of Aβ [38]. Several reports have provided evidence demonstrating a role for IL-1β in the etiology of AD based largely on the finding that IL-1β expression in different brain areas in AD and also in the cerebrospinal fluid of AD patients [39,40]. Consistently, the injection of Aβ increases the hippocampal mRNA expression of both TNF-α and inducible nitric oxide synthase (iNOS), of which the former was stronger, and the knock-out of TNF-α (TNF-α (−/−)) in mouse prevented the increase of iNOS mRNA in the hippocampus and the impairment of recognition memory in mice induced by Aβ [41]. It has been proposed that elevated levels of pro-inflammatory cytokines, including TNF-α, may inhibit phagocytosis of Aβ in AD brains thereby hindering efficient plaque removal by resident microglia [42]. In our studies, we demonstrated that the hippocampus of the Aβ_1-40_ group had the activation of astrogliosis and microgliosis as well as the strong increase of IL-1β and TNF-α compared with the sham group, indicating that the two inflammatory cytokines were involved in the inflammatory response in AD rats.

Our results demonstrated that NaHS treatment decreased Aβ_1-40_-induced astrocytic and microglial response as well as inflammatory cytokines expression. A previous study showed that H_2_S was synthesized in brain primarily by the enzyme CBS, and that astrocytes were the most active producers of H_2_S, with much smaller quantities being generated by microglia [43]. H_2_S production is suppressed by inflammatory stimulation of microglia and astrocytes, and this suppression reduces the natural anti-inflammatory effect of H_2_S [14]. H_2_S has been reported to exhibit marked anti-inflammatory activity in LPS-induced lung, liver, and kidney tissue inflammatory damage in the mouse [44]. Another result reveals that H_2_S releasing NSAIDS S-aspirin and S-diclofenac attenuates the neuroinflammation induced by activation of glia [45]. Administration of NaHS significantly attenuates LPS-induced cognitive impairment through reducing the overproduction of proinflammatory mediators, and accompanied by an increase of H_2_S levels [46]. However, there is no direct evidence that H_2_S attenuates inflammatory initiated neuronal death, which is closely associated with the pathogenesis of several neurodegenerative diseases including AD.

To further understand the molecular mechanisms of the effects of NaHS on the expressions of IL-1β and TNF-α, the expressions of phospho-p38 MAPK, phospho-p65 NF-κB, and phospho-JNK were analyzed by western blot. Numerous studies demonstrate that injection of Aβ into the hippocampus, cortex, and nucleus basalis induces the activity of p38 MAPK, NF-κB, and JNK in rat [47-50]. p38 MAPK activation has been implicated in the pathogenesis of AD, and significant increase of MAPK kinase 6 (MKK6), one of the upstream activators of p38 MAPK, is observed in hippocampal and cortical regions of individuals with AD compared with age-matched controls [51]. Chronic exposure of human microglia to Aβ_1-42_ led to enhanced p38 MAPK expression [36]. NF-κB is known to upregulate the expressions of cytokines, chemokines, adhesion molecules, acute phase proteins, and inducible effector enzymes. NF-κB is composed of several protein subunits, among which p65 has been extensively studied. In AD brains, p65 NF-κB immunoreactivity is greater in neurons and astrocytes surrounding amyloid plaques [52,53]. Additionally, it has been reported that Aβ stimulation leads to p65 NF-κB activation in cultured neurons and glia [54]. One study has revealed that increased hippocampal IL-1βconcentration, paralleled by increased JNK activation in AD brain [51]. The activation of JNK has been described in cultured neurons after Aβ exposure, and their inhibition attenuates Aβ toxicity [49,55]. Corroborating these findings, the present results show that increased hippocampal concentration of inflammatory cytokines stimulated by Aβ is accompanied by phosphorylation of p38 MAPK, p65 NF-κB, and JNK.

Our data also suggest that the effects of H_2_S against the released levels of pro-inflammatory cytokines such as IL-1β and TNF-α may include the capability of this gas to reduce the levels of phospho-p38 MAPK and phospho-p65 NF-κB but not phospho-JNK. This is consistent with reports that S-diclofenac decreases the activation of NFκB and other pro-inflammatory cytokines in rat plasma and liver homogenates [44] and that NaHS attenuates LPS-induced inflammation by inhibition of p38 MAPK and p65 NF-κB in rodent microglia and rat [12,46].

## Conclusions

In conclusion, our results clearly demonstrated that: (1) a single injection of Aβ_1-40_ into the hippocampus produced cognitive impairment in rats, apoptosis, and the glial response, with concomitant production of IL-1β and TNF-α, and these effects occurred via activation of p38 MAPK, p65 NF-κB, and phospho-JNK in rat’s hippocampus; and (2) pretreatment with NaHS significantly attenuated Aβ_1-40_-induced cognitive deficits, apoptosis, and the glial response, with concomitant inhibitions of IL-1β and TNF-α production, as well as repressed Aβ_1-40_-induced activation of p38 MAPK and p65 NF-κB.

## Competing interests

The authors declare that they have no competing interests.

## Authors’ contributions

AX designed the study, conducted molecular assays and the data analysis. DL participated in the design of the study. JIANL performed the TUNEL assay. WJ carried out the RT-PCR. MZ performed the immunohistochemistry. LH performed the Morris water maze. JIL participated in the ELISA and performed the statistical analysis. AX, DL, WJ, MZ, LH, and JIANL drafted and/or criticized the manuscript. All authors read and approved the final manuscript.
